# Systemic Immune-Inflammation Index and Its Association with the Prevalence of Stroke in the United States Population: A Cross-Sectional Study Using the NHANES Database

**DOI:** 10.31083/j.rcm2504130

**Published:** 2024-04-02

**Authors:** Guangcheng Liu, Hao Qian, Liang Wang, Wei Wu

**Affiliations:** ^1^Department of Cardiology Medicine, Peking Union Medical College Hospital, Chinese Academy of Medical Science and Peking Union Medical College, 100006 Beijing, China

**Keywords:** SII, stroke, prediction, NHANES

## Abstract

**Background:**

The purpose of this study was to evaluate the ability of the systemic immune-inflammation index (SII) to predict the prevalence of stroke in the American population.

**Methods:**

A cross-sectional research study of 53,600 people was carried out utilizing information from the U.S. National Health and Nutrition Examination Survey (NHANES) database. Participants were divided into three groups based on the tertiles of their SII levels: SII-low, SII-median, and SII-high. Logistic regression analysis was used to investigate SII and the prevalence of stroke. Subgroup analyses, sensitivity analyses, and restricted cubic spline (RCS) analysis were also carried out.

**Results:**

A total of 2368 patients with stroke were found among the participants in this cross-sectional study. The high SII group had a substantially greater prevalence of stroke compared to the low SII group (odds ratio [OR] = 1.18, 95% confidence interval [CI] 1.01, 1.42). The risk of stroke decreased by 34% for every unit rise in log-transformed SII (OR 1.30, 95% CI 0.99, 1.70). A positive linear connection between SII levels and the prevalence of stroke was revealed using RCS analysis (*p* for non-linearity = 0.387).

**Conclusions:**

This cross-sectional study utilizing large-scale data from NHANES provides the first evidence of a significant association between higher SII levels and increased prevalence of stroke. These findings highlight the relevance of SII as a potential predictive marker for stroke.

## 1. Introduction

Stroke is a significant global public health problem characterized by high morbidity and mortality rates [1, 2]. The alarming number of reported strokes and associated deaths, along with escalating medical costs, highlight the need to identify individuals who are at high risk of stroke [2, 3]. Inflammation has been recognized as a crucial factor in the development of cerebral vessel disease [4]. The systemic immune-inflammation index (SII) is an immune-based biomarker derived from platelet, neutrophil, and lymphocyte counts. The SII has shown promise in predicting various diseases, including cardiovascular diseases [5], cancers [6], hepatic steatosis [7], osteoporosis [8], and diabetic kidney disease [9]. Previous studies that explored the relationship between SII and stroke reported a strong association between elevated SII levels and increased stroke incidence in the general population, and with unfavorable outcomes in stroke patients [5, 10]. Notably, a recent cohort study on Chinese adults found a significant association between high log-transformed SII levels and the risk of total stroke and ischemic stroke [5]. Additionally, a meta-analysis of retrospective studies confirmed the link between high SII levels and poor stroke outcomes [10]. However, the predictive value of high SII for stroke incidence in individuals from the United States has yet to be validated. Therefore, in the present study we analyzed data on 57,600 participants obtained from the U.S. National Health and Nutrition Examination Survey (NHANES) to investigate whether SII has predictive value for stroke incidence in the U.S. population.

## 2. Methods

### 2.1 Study Design and Participants

**Fig. 1.**
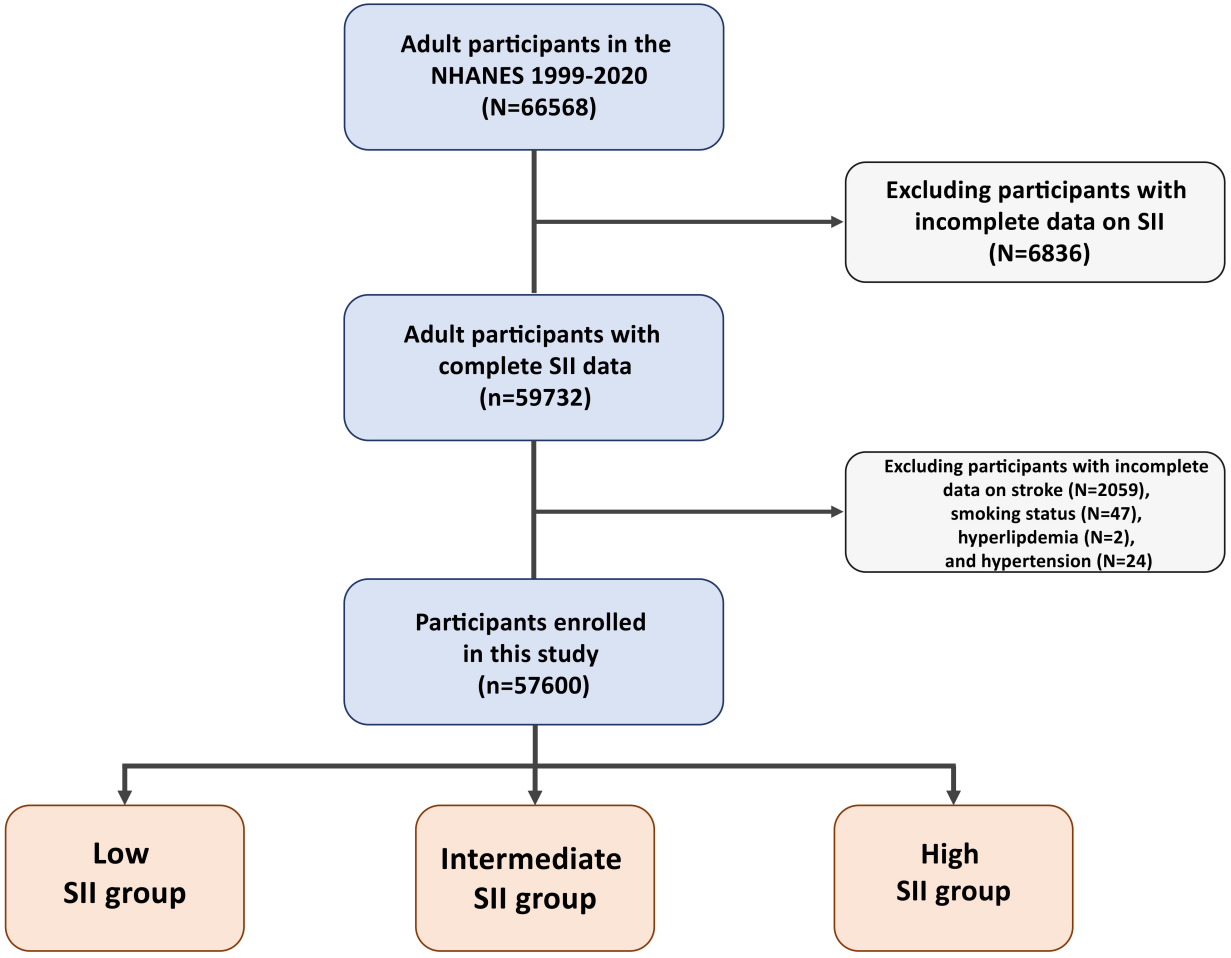
**Flowchart of participant selection**. SII, systemic immune-inflammation index; NHANES, National Health and Nutrition Examination Survey.

This study included adults aged 18 or above in the NHANES database and spanning the years from 1999 to 2020. Initially, a total of 66,568 adult participants were enrolled. Participants were excluded if they lacked information on SII data (n = 6836) or had missing data on stroke diagnosis (n = 2059), smoking status (n = 47), hyperlipidemia (n = 2), and hypertension (n = 24). The final analysis included 57,600 participants, of which 2368 were stroke patients (refer to Fig. 1 for the participant flowchart). The participants were categorized into three groups based on SII tertiles: SII-low (<384), SII-median (384–597), and SII-high (≥597). The primary aim of this study was to examine the association between SII and the prevalence of stroke in the entire population.

SII was calculated using the formula: (platelet count × neutrophil count)/lymphocyte count. Lymphocyte, neutrophil, and platelet counts were measured using automated hematology analyzers and expressed as × ${\mathrm{10}}^{3}$ cells/mL [11].

The Ethics Review Board of the National Center for Health Statistics (NCHS) of the Centers for Disease Control and Prevention approved the database protocols, and all participants provided written informed consent before enrollment.

Participants with incomplete data for SII (n = 6836), stroke (n = 2059), smoking status (n = 47), hyperlipdemia (n = 2) or hypertension (n = 24) were excluded, giving a final total of 57,600 participants from the initial NHANES group (n = 66,568). Participants were categorized into three groups according to SII tertiles: SII-low (<384), SII-median (384–597), and SII-high (≥597).

### 2.2 Definitions

The primary outcome measure in this study was self-reported stroke, as determined by asking participants: “Have you ever been told you had a stroke?”, or “Has a doctor or other health professional ever told you that you had a stroke?”. Diabetes mellitus was defined based on self-reported medical history, use of oral hypoglycemic agents or insulin, fasting glucose level ≥126 mg/dL, or hemoglobin A1c level ≥6.5% [12]. Hyperlipidemia was defined as serum triglycerides ≥150 mg/dL, total cholesterol ≥200 mg/dL, low-density lipoprotein cholesterol ≥130 mg/dL, high-density lipoprotein cholesterol ≤40 mg/dL in men or ≤50 mg/dL in women, or use of medication for hyperlipidemia [13]. The presence of cancer was determined based on the self-reported question: “Have you ever been told you had cancer or malignancy?”. The biochemical parameters mentioned above were measured in a subset of participants who provided blood samples at the Mobile Examination Center (MEC).

### 2.3 Statistical Analysis

In this study, appropriate weights were employed to account for the complex sampling design of NHANES, thereby ensuring a representative sample of the US national population (https://www.cdc.gov/nchs/nhanes/index.htm). Baseline characteristics were reported as weighted means ± standard error for continuous variables, and frequency (weighted percentages) for categorical variables. Differences between groups were compared using Analysis of Variance (ANOVA) for continuous variables, and chi-square tests for categorical variables. The percentage of missing data for covariates was <5% (body mass index (BMI) [1.72%]), except for the family income to poverty ratio (9.4%). Missing values for the family income to poverty ratio were categorized as “Unknown”. To incorporate all available data for modeling, imputation with the median of each variable was performed.

For the analysis, odds ratios (ORs) and 95% confidence intervals (CIs) were estimated using multivariate logistic regression models to assess the association between SII and the prevalence of stroke. When treating SII as a continuous variable, a log-transformation was applied and the change in stroke prevalence for each one-unit increase in log-transformed SII was determined. Restricted cubic spline (RCS) analysis in the fully adjusted model was employed to evaluate the dose-response relationship between SII and stroke, with nonlinearity assessed using the likelihood ratio test.

Covariates were progressively adjusted in three models for logistic regression analysis. Model 1 included adjustments for age (continuous), sex (male or female), and race/ethnicity (non-Hispanic White, non-Hispanic Black, Mexican American, and others). Model 2 expanded on Model 1 by further adjusting for smoking status (never, former, current), physical activity (sedentary, insufficient, moderate, high), education level (under high school, high school or equivalent, college or higher), family income to poverty ratio (≤1.0, 1.0–3.0, >3.0, unknown), and BMI (<25.0, 25.0–29.9, ≥30.0 ${\mathrm{kg/m}}^{2}$). Model 3 (fully adjusted model) incorporated all the covariates from Model 2, as well as diabetes, hyperlipidemia, and hypertension. **Supplementary Method 1** provides additional details on covariate assessment.

Subgroup analysis was conducted based on age (<60 or ≥60 years), sex (male or female), race/ethnicity (White or non-White), smoking status (never or former/current), BMI (<30.0 or ≥30.0 ${\mathrm{kg/m}}^{2}$), physical activity (sedentary/insufficient or moderate/high), hyperlipidemia (yes or no), hypertension (yes or no), and diabetes (yes or no). The significance of interaction terms between the stratification variables and SII was evaluated using the Wald test. Sensitivity analyses were performed by excluding non-Hispanic Black participants and those with missing data on BMI in the fully adjusted model.

All statistical analyses were conducted using R version 4.1.3 (R Foundation for Statistical Computing, Vienna, Austria) with the “survey” package. A two-tailed *p*-value < 0.05 was considered statistically significant.

## 3. Results

### 3.1 Characteristics of the Study Population

The baseline characteristics of all participants, categorized by SII levels, are presented in Table 1. The overall weighted mean age of the 57,600 participants included in the analysis was 47.48 years, and 51.86% were males. Individuals with SII-high tended to be older, male, non-Hispanic white, nonsmokers, with higher levels of education, family income, BMI, waist circumference, and physical activity, and lower levels of estimated glomerular filtration rate (eGFR). The prevalence of diabetes, hypertension, hyperlipidemia, cancer, and stroke was significantly lower in the SII-low group compared to the SII-high group.

**Table 1.** **Baseline characteristics of participants from NHANES according to SII**.

Characteristics		Total (N = 57,600)	SII			*p* value
			Low (N = 19,212)	Median (N = 19,188)	High (N = 19,200)	
Age (years)		47.48 ± 0.19	46.73 ± 0.25	47.38 ± 0.21	48.29 ± 0.24	<0.001
Sex, n (%)						<0.001
	Male	29,870 (51.86)	8948 (46.17)	9905 (51.68)	11,017 (57.78)	
	Female	27,730 (48.14)	10,264 (53.83)	9283 (48.32)	8183 (42.22)	
Race/ethnicity, n (%)						0.02
	Non-Hispanic White	1707 (57.17)	460 (70.46)	464 (73.11)	783 (79.55)	
	Non-Hispanic Black	668 (22.37)	229 (17.91)	203 (14.69)	236 (9.82)	
	Mexican American	456 (15.27)	119 (4.92)	126 (5.40)	211 (5.21)	
	Others	155 (5.19)	39 (6.71)	48 (6.80)	68 (5.42)	
Education level, n (%)						<0.001
	Less than high school	14,960 (25.97)	5056 (16.43)	4960 (15.32)	4944 (16.02)	
	High school or equivalent	13,379 (23.23)	4307 (22.64)	4424 (24.26)	4648 (25.26)	
	College or above	29,261 (50.8)	9849 (60.93)	9804 (60.42)	9608 (58.72)	
Family income to poverty ratio, n (%)						0.003
	<1	10,721 (18.61)	3551 (12.94)	3517 (12.57)	3653 (13.34)	
	≥1 & <3	22,019 (38.23)	7310 (33.46)	7148 (31.89)	7561 (34.39)	
	≥3	19,443 (33.76)	6445 (46.07)	6754 (48.11)	6244 (44.92)	
	Unknown	5417 (9.4)	1906 (7.53)	1769 (7.43)	1742 (7.35)	
Smoking status, n (%)						<0.001
	Never	31,608 (54.87)	10,947 (57.29)	10,623 (55.54)	10,038 (51.57)	
	Former	14,225 (24.7)	4622 (24.85)	4679 (24.32)	4924 (25.35)	
	Current	11,767 (20.43)	3643 (17.86)	3886 (20.14)	4238 (23.08)	
BMI (${\mathrm{kg/m}}^{2}$), n (%)						<0.001
	<25.0	16,401 (28.47)	5801 (32.51)	5329 (28.38)	5271 (27.72)	
	25.0–29.9	20,015 (34.75)	6865 (35.22)	6747 (34.45)	6403 (31.89)	
	≥30.0	21,184 (36.78)	6546 (32.26)	7112 (37.17)	7526 (40.39)	
Physical activity, n (%)						<0.001
	Sedentary	15,967 (27.72)	4940 (19.49)	5168 (21.41)	5859 (24.97)	
	Insufficient	11,890 (20.64)	3556 (16.36)	3984 (18.51)	4350 (21.01)	
	Moderate	6570 (11.41)	2108 (10.80)	2239 (11.64)	2223 (12.41)	
	High	23,173 (40.23)	8608 (53.35)	7797 (48.44)	6768 (41.60)	
Diabetes, n (%)		10,335 (17.94)	3389 (12.60)	3336 (13.45)	3610 (15.67)	<0.001
Hyperlipidemia, n (%)		40,996 (71.17)	13,015 (65.61)	13,919 (71.47)	14,062 (72.17)	<0.001
Hypertension, n (%)		23,918 (41.52)	7742 (33.99)	7801 (36.04)	8375 (40.65)	<0.001
Cancer, n (%)		5351 (9.3)	1543 (8.95)	1683 (9.31)	2125 (11.40)	<0.001
Stroke, n (%)		2368 (4.11)	676 (2.58)	755 (2.97)	937 (3.72)	<0.001

Data are presented as weighted means ± SEs for continuous variables, and as unweighted numbers (weighted percentages) for categorical variables. Abbreviations: BMI, body mass index; SII, systemic immune-inflammation index; NHANES, National Health and Nutrition Examination Survey; N, number.

### 3.2 Association of SII with the Prevalence of Stroke

**Fig. 2.**
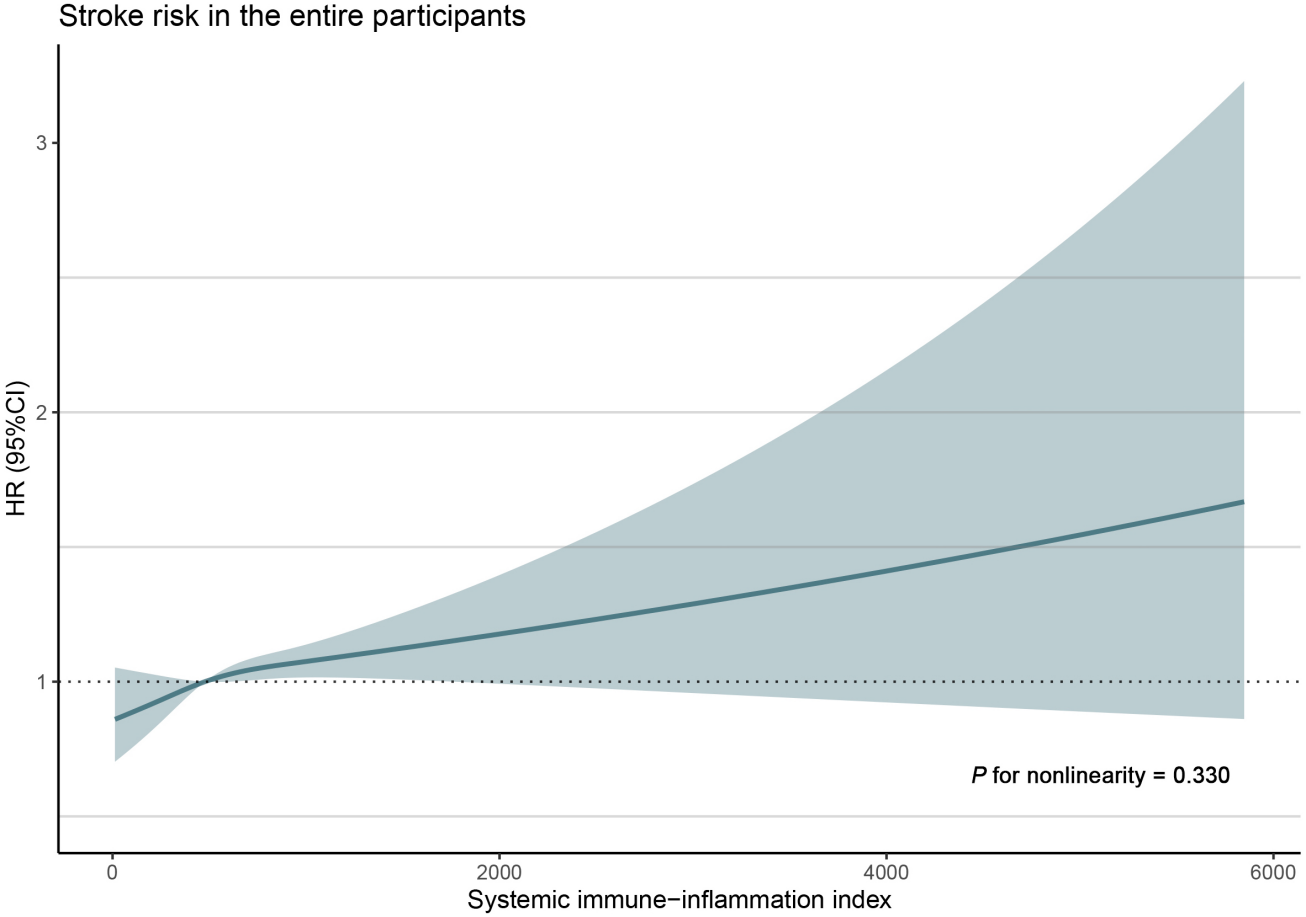
**Restricted cubic spline model for the association of SII with the prevalence of stroke**. RCS analysis demonstrated a positive linear correlation between SII level and the prevalence of stroke (*p* for non-linearity = 0.387) in the overall participant group. SII, systemic immune-inflammation index; RCS, restricted cubic spline; CI, confidence interval; HR, hazard ratio.

The association between stroke and SII, analyzed as a continuous or categorical variable, is presented in Table 2. The results indicate that higher SII levels are associated with an increased likelihood of stroke. When SII was analyzed as a continuous variable, each unit increase in log-transformed SII was associated with a 34% higher risk of stroke in Model 2 (OR 1.34, 95% CI 1.02, 1.76). A statistically significant association was not observed with Model 3 when SII was analyzed as a continuous variable, however the trend was consistent with Model 2 as *p* trend < 0.05. When SII was examined as a categorical variable, participants in the SII-high group had a significantly higher prevalence of stroke compared to those in the SII-low group (OR 1.18, 95% CI 1.01, 1.42). RCS analysis was also performed to evaluate the relationship between SII levels and stroke risk in the overall participant group. The analysis demonstrated a positive linear correlation between SII level and the prevalence of stroke (*p* for non-linearity = 0.387), as shown in Fig. 2.

**Table 2.** **Logistic regression analysis for the risk of stroke according to SII in the overall NHANES participant group**.

Model	Per one unit increase in log-transformed SII OR (95% CI)	OR (95% CI)			
		Low	Median	High	*p* trend
Crude	1.98 (1.47, 2.66)	1.00	1.16 (0.97, 1.37)	1.46 (1.23, 1.74)	<0.001
Model 1	1.54 (1.17, 2.03)	1.00	1.14 (0.96, 1.36)	1.32 (1.11, 1.58)	0.002
Model 2	1.34 (1.02, 1.76)	1.00	1.11 (0.93, 1.33)	1.22 (1.02, 1.47)	0.028
Model 3	1.30 (0.99, 1.70)	1.00	1.10 (0.92, 1.31)	1.18 (1.01, 1.42)	0.041

Model 1: adjusted for age, sex, and race/ethnicity; Model 2: further adjusted (from Model 1) for smoking status, physical activity, education level, family income to poverty ratio, and BMI; Model 3: further adjusted (from Model 2) for diabetes, dyslipidemia, cancer, and hypertension. OR, odds ratio; CI, confidence interval; BMI, body mass index; SII, systemic immune-inflammation index; NHANES, National Health and Nutrition Examination Survey.

### 3.3 Subgroup and Sensitivity Analysis

The results of subgroup analyses are presented in Table 3. Similar trends were observed across different subgroups, including age (<60 or ≥60 years), sex (male or female), race/ethnicity (non-Hispanic White or other), smoking status (never or former/current), BMI (<30 or ≥30 ${\mathrm{kg/m}}^{2}$), physical activity (sedentary/insufficient or moderate/high), dyslipidemia (yes or no), hypertension (yes or no), and diabetes (yes or no). No significant interactions were found between the ORs and these stratifying variables, except for sex. This suggests that male participants tended to have higher SII levels and a higher likelihood of developing stroke.

**Table 3.** **Subgroup analyses of the associations between SII and stroke in the overall participant cohort**.

Subgroup		No. Stroke/Total	OR (95% CI)			*p *for interaction
			Low	Median	High	
Age (years)						0.368
	<60	1757/37,767	1.00	0.98 (0.72, 1.33)	1.25 (0.91, 1.70)	
	≥60	611/19,833	1.00	1.19 (0.98, 1.45)	1.20 (0.99, 1.45)	
Sex						0.029
	Male	1174/29,870	1.00	1.34 (1.06, 1.69)	1.21 (0.95, 1.52)	
	Female	1194/27,730	1.00	0.94 (0.76, 1.16)	1.19 (0.94, 1.51)	
Race/ethnicity						0.210
	White People	1173/24,872	1.00	1.06 (0.84, 1.35)	1.13 (0.88, 1.45)	
	Non-White People	1195/32,728	1.00	1.27 (1.03, 1.56)	1.54 (1.26, 1.89)	
Smoking status						0.763
	Never	965/31,608	1.00	1.15 (0.91, 1.45)	1.30 (1.01, 1.67)	
	Former/Current	1403/25,992	1.00	1.09 (0.86, 1.37)	1.18 (0.94, 1.47)	
BMI, ${\mathrm{kg/m}}^{2}$						0.650
	<30	1411/36,416	1.00	1.13 (0.92, 1.39)	1.24 (0.98, 1.58)	
	≥30	957/21,184	1.00	1.07 (0.83, 1.39)	1.10 (0.86, 1.41)	
Physical activity						0.446
	Sedentary/Insufficient	1556/27,857	1.00	1.10 (0.88, 1.38)	1.10 (0.89, 1.36)	
	Moderate/High	812/29,743	1.00	1.08 (0.82, 1.42)	1.31 (0.98, 1.75)	
Dyslipidemia						0.803
	Yes	1987/40,996	1.00	1.09 (0.90, 1.32)	1.18 (0.97, 1.44)	
	No	381/16,604	1.00	1.15 (0.77, 1.71)	1.35 (0.88, 2.06)	
Hypertension						0.464
	Yes	1896/23,918	1.00	1.07 (0.89, 1.29)	1.21 (1.01, 1.45)	
	No	472/33,682	1.00	1.18 (0.80, 1.72)	1.04 (0.70, 1.56)	
Cancer						0.384
	Yes	480/5351	1.00	0.99 (0.74, 1.33)	1.28 (0.92, 1.77)	
	No	4871/52,200	1.00	1.14 (0.95, 1.38)	1.19 (0.98, 1.45)	
Diabetes						0.041
	Yes	929/10,335	1.00	1.35 (1.02, 1.78)	1.14 (0.89, 1.47)	
	No	1439/47,265	1.00	0.99 (0.80, 1.22)	1.22 (0.97, 1.53)	

All the models were adjusted for age, sex, race/ethnicity, smoking status, physical activity, education level, family income to poverty ratio, BMI, diabetes, dyslipidemia, cancer, and hypertension. OR, odds ratio; CI, confidence interval; BMI, body mass index; SII, systemic immune-inflammation index.

Table 4 provides a summary of the sensitivity analyses conducted to assess the associations between SII and the prevalence of stroke. The results remained consistent even after excluding non-Hispanic Black participants and participants with missing data on BMI.

**Table 4.** **Sensitivity analyses of the associations (OR, 95% CI) between SII and prevalence of stroke in the overall NHANES participant cohort**.

Analysis	Low	Median	High	*p* trend
Excluding non-Hispanic Black participants (N = 45,428)	1.00	1.13 (0.92, 1.40)	1.23 (1.01, 1.52)	0.049
Excluding participants with missing data on BMI (N = 56,610)	1.00	1.11 (0.93, 1.34)	1.14 (1.04, 1.39)	0.047

All models were adjusted for age, sex, race/ethnicity, smoking status, physical activity, education level, family income to poverty ratio, BMI, diabetes, dyslipidemia, cancer, and hypertension. OR, odds ratios; CI, confidence interval; BMI, body mass index; SII, systemic immune-inflammation index; NHANES, National Health and Nutrition Examination Survey; N, number.

## 4. Discussion

This study is the first attempt to investigate the relationship between SII and the incidence of stroke using data from the NHANES database. A comprehensive analysis was conducted on a total of 53,111 participants, providing valuable insights into the association between SII levels and the prevalence of stroke. The findings revealed that higher SII levels were linked to a higher prevalence of stroke, indicating a significant positive correlation. This association followed a dose-response pattern, further strengthening the evidence.

Previous studies have examined the association between SII and stroke. Xu *et al*. [5] reported results from a prospective cohort study in 2021 involving 13,929 adults. These authors found that high SII was associated with an increased risk of total stroke and ischemic stroke, suggesting the potential of SII as a biomarker for stroke incidence [5]. A subsequent cohort study in a Chinese population with 85,153 participants provided further support for this finding by demonstrating a positive dose-response relationship between SII and stroke risk [14]. A meta-analysis of 13 studies also confirmed the predictive value of high SII for an increased incidence of ischemic and hemorrhagic stroke [15]. Whereas these studies focused on the Chinese population, the aim of the current work was to validate the link between SII and stroke risk in the U.S. population by analyzing a large sample from NHANES. As expected, high SII levels were found to be associated with a higher prevalence of stroke in the U.S. population.

High SII also shows prognostic value in stroke patients. A recent meta-analysis of 19 retrospective studies indicated that high SII was associated with significantly poorer outcomes in terms of increased mortality and a higher incidence of hemorrhagic transformation [10]. Another cross-sectional study reported that elevated log-transformed SII levels were associated with an increased risk of atrial fibrillation in stroke patients, highlighting the need for close monitoring of these individuals [16]. While the prognostic value of high SII in stroke patients was not evaluated in the present study, future investigations using the NHANES database should aim to validate this conclusion.

The significant cerebral toxicity associated with high SII can be attributed to increased inflammation and a prothrombotic state. The platelet-leukocyte interaction is considered a key contributor and involves the exchange of signals between platelets and various types of leukocytes [5]. This process promotes atherothrombosis and inflammatory immune reactions, thereby exacerbating endothelial injury and atherosclerosis. The delivery of RANTES and platelet factor-4 amplifies monocyte recruitment, leading to inflammation and atherosclerosis [17]. In addition to leukocytes, the recruitment of neutrophils was suggested to exacerbate endovascular injury, leading to thrombosis and increasing the risk of thrombotic complications including stroke [18]. The recruitment of neutrophils is mediated by activated platelets via *p*-selectin and β_2_/β_3_-integrin receptors, or binding to the triggering receptor expressed on myeloid cells-1 (TREM-1) receptor on the neutrophil surface [19].

The strength of this study lies in the use of a large sample from the NHANES database to comprehensively analyze the association between SII and stroke. By including various demographic and clinical factors, subgroup analyses were able to assess the consistency of the findings across different population groups. Furthermore, sensitivity analyses were conducted to verify the robustness of the results, yielding consistent outcomes even after excluding certain participants.

However, several limitations should be acknowledged. The cross-sectional design of this study prevents the identification of causal relationships, highlighting the need for longitudinal studies to further explore the impact of SII on stroke incidence. Moreover, the reliance on self-reported data for stroke diagnosis in the absence of medical records or imaging verification introduces the possibility of misclassification or underreporting. Future studies should therefore aim to incorporate more rigorous diagnostic measures. Additionally, the moderate sample size of this study might have restricted the statistical power and generalizability of the results. Larger studies are warranted to strengthen the conclusions and enhance their applicability to broader populations.

## 5. Conclusions

In summary, this study provides important insights into the association between SII and stroke incidence in the American population based on the NHANES database. Our findings support a positive correlation between higher SII levels and an increased prevalence of stroke, consistent with previous research conducted primarily in the Chinese population. The mechanisms underlying this association are likely to involve inflammatory and thrombotic processes. However, given the study’s limitations, further research using longitudinal study designs and more rigorous design methods (such as excluding regional limitation) is needed to validate these findings and to confirm the prognostic value of SII in stroke patients. Ultimately, a better understanding of the relationship between SII and stroke may contribute to improved risk assessment and targeted interventions for stroke prevention and management.

## Data Availability

All data are available at the NHANES website https://www.cdc.gov/nchs/nhanes/index.htm.

## Supplementary Material

Supplementary material associated with this article can be found, in the online version, at https://doi.org/10.31083/j.rcm2504130.
