# Supplementary material for: Systemic Immune-Inflammation Index and Its Association with the Prevalence of Stroke in the United States Population: A Cross-Sectional Study Using the NHANES Database
Source: Rev Cardiovasc Med. 2024 Apr 2;25(4):130. doi: 10.31083/j.rcm2504130 (PMC11264029; doi:10.31083/j.rcm2504130)
Supplement: Supplementary file 1 [file 2153-8174-25-4-130-s1.docx]

**Supplementary method 1. Assessment of covariates**

Information on age, sex, race/ethnicity, smoking status, physical activity, and self-reported medical conditions were obtained through standardized questionnaires during in-home interviews by trained interviewers. Heights, weights, waist circumferences, blood pressures, and blood samples were collected from physical examinations at MEC using standard protocols.

Race/ethnicity was categorized as non-Hispanic White people, non-Hispanic Black people, Mexican American people, and others. Education level was categorized as under high school, high school or equivalent, and college or higher. The family income to poverty ratio was classified as ≤ 1.0, 1.0 –3.0, and > 3.0. Physical activity was measured as the weekly minutes of moderate and vigorous activities multiplied by the metabolic equivalent (MET) level and divided into four categories: sedentary (without regular physical activity, MET-minutes/week = 0), insufficient (0 < MET-minutes/week < 500), moderate (500 ≤ MET-minutes/week ≤ 1000), and high (>1000 MET-minutes/week) (1). Smoking status was categorized as never (smoked less than 100 cigarettes in life), former (smoked more than 100 cigarettes in life but quit smoking now), and current (smoked more than 100 cigarettes in life and still smoke some days or every day). Body mass index (BMI, kg/m^2^) was calculated as weight in kilograms divided by height in meters squared. Diabetes mellitus, hyperlipidemia, cancer, and hypertension were presented as “yes” or “no”.

Reference

1. Third Report of the National Cholesterol Education Program (NCEP) Expert Panel on Detection, Evaluation, and Treatment of High Blood Cholesterol in Adults (Adult Treatment Panel III) final report. Circulation (2002);106(25):3143-421.
